# GD2-CAR NK-92 cell activity against neuroblastoma cells is insusceptible to TIGIT knockout

**DOI:** 10.1007/s00262-025-04010-6

**Published:** 2025-05-03

**Authors:** Wiebke Jünemann, Isabelle Bley, Laura Rekowski, Marianne Klokow, Susanne Herppich, Ingo Müller, Kerstin Cornils

**Affiliations:** 1 Children’s Cancer Centre Research Institute Hamburg, Hamburg, Germany; 2 Division of Paediatric Stem Cell Transplantation and Immunology, Department of Paediatric Haematology and Oncology, University Medical Centre Hamburg-Eppendorf, Hamburg, Germany

**Keywords:** Neuroblastoma, Immune checkpoint, Immunotherapy, Chimeric antigen receptor, PVR, PVRL2, TIGIT

## Abstract

Immunotherapy by inhibition of immune checkpoint (IC) molecules has emerged as an important cancer therapy. Among these lC, the poliovirus receptor/poliovirus receptor-like 2 protein (PVR/PVRL2)-TIGIT axis was discovered as potential target for various cancers. For neuroblastoma (NB), the most common extracranial solid cancer in children, no effective IC therapy has been established yet. To investigate the PVR/PVRL2-TIGIT IC axis as a new target for the treatment of NB, we analysed whether PVR and PVRL2 influence the survival of patients and verified the expression of the receptors on NB cell lines. To disrupt the checkpoint axis, we performed single and double knockouts of these receptors on NB cell lines and subsequently removed TIGIT, an inhibitory receptor on immune effector cells, from NK-92 cells. Finally, we combined checkpoint inhibition with GD2-CAR NK-92 cells and investigated changes in cytotoxicity. Using RNA-Seq data we showed that the expression of PVR and PVRL2 on NB cells correlates to a lower event-free survival of patients. CRISPR/Cas9 knockouts of PVR and PVRL2 showed no improved cytotoxic activity of NK-92 cells. We observed enhanced lysis of NB cells using TIGIT-deficient NK-92 cells. However, the cytotoxicity of GD2-CAR NK-92 was not significantly enhanced. In summary, we have shown that in addition to the interaction of PVR/PVRL2 and TIGIT on engineered immune effector cells against NB, pleiotropic ligands appear to be relevant. Deletion of TIGIT from immune effector cells is a promising approach to protect these cells from tumour-associated inhibitory signals but cannot enhance the effect of GD2-CAR-NK-92 cells.

**Supplementary Information:**

The online version contains supplementary material available at 10.1007/s00262-025-04010-6.

## Introduction

Neuroblastoma (NB) is the most common extracranial solid tumour in children and accounts for more than 8-10% of malignancies in patients younger than 15 years [1] with poor survival rates (< 45%) for high-risk patients [2]. Additionally, 50-60% of patients develop a recurrent disease after treatment associated with a 5-year survival rate of less than 20% [3]. Current treatments for high-risk NB include immunotherapy with Dinutuximab, a monoclonal antibody targeting ganglioside GD2 on the surface of NB cells [4]. Although this treatment option currently shows promising results and is established as a standard therapy, its success is limited and comes with serious side effects like neuropathic pain [5]. In comparison, a chimeric antigen receptor (CAR) T cell therapy against GD2 did not show these side effects in NB patients [6] and revealed a significantly improved outcome for relapsed or refractory high-risk NB with a 3-year overall survival of 60% and event-free survival of 36% [7].

To further improve these promising results, it is important to understand the interaction between tumour cells and immune cells. Tumour cells create an immunosuppressive microenvironment through the presence of ligands on their surfaces, which hamper the function of immune cells (e.g. [8]). One of the major immune evasion mechanisms is the presentation of ligands binding to inhibitory checkpoint proteins on immune cells, thereby inhibiting immune cell activation and sufficient anti-cancer immune responses [9]. In the past decade, immune checkpoint (IC) inhibitor therapies targeting checkpoint molecules or their ligands with monoclonal antibodies have been developed. For example, blocking the negative IC molecules cytotoxic T-lymphocyte-associated protein 4 (CTLA-4) [10] or the programmed cell death protein 1 and its ligand 1 (PD-1/PDL-1) [11] has been shown to reactivate cytotoxic immune cells and support efficient tumour rejection. This strategy has evolved into an important treatment option for numerous types of cancer. Since only a subset of cancer patients responds to these therapies, the evaluation of other relevant IC axes is still relevant [12].

Recently, the IC axis involving the poliovirus receptor (PVR, CD155), poliovirus receptor-like 2 (PVRL2, CD112), and T cell immunoreceptor with Ig and ITIM domains (TIGIT) has emerged as a promising target for cancer immunotherapy [13]. Its relevance has already been proven for various malignancies such as e.g. acute myeloid leukaemia [14]. Importantly, TIGIT was described also as a potential new immunotherapeutic target in NB [15, 16]. Interaction of TIGIT with PVR or PVRL2 on tumour cells leads to the inhibition of immune cells via the immunoreceptor tyrosine-based inhibitory motif (ITIM) domain. In NK cells, this inhibition preserves their self-tolerance but also hampers cytotoxicity against tumour cells, which promotes cancer cell survival [17]. However, the interaction of PVR and PVRL2 with DNAX accessory molecule 1 (DNAM-1, CD226), an activating receptor, leads to coactivation and lysis of PVR/PVRL2 presenting cells [17]. It is therefore crucial which specific receptors interact, as either immune cell activation or inhibition is triggered. In tumour-infiltrating NK cells, it was shown that TIGIT is upregulated, leading to an inhibitory phenotype of the cells [16].

NK cells are potent cytotoxic effector cells, which have become increasingly used in immunotherapeutic approaches. They belong to the innate immune system and provide a rapid immune response against cancer cells without specific antigen presentation. To gain specificity, NK cells can additionally be equipped with a CAR to generate CAR-NK cells, which have already been used in clinical trials against haematological malignancies and (metastatic) solid tumours [18]. However, the usage of primary NK (pNK) cells is mostly impaired by long expansion protocols to reach sufficient cell numbers, accompanied by a reduction of cytotoxicity and a low transduction efficiency. As an alternative, the NK cell line NK-92 is available, which shows phenotypic and functional characteristics of activated pNK cells. Irradiated NK-92 cells still exhibit cytotoxic activity and were already proven safe in clinical trials [19]. The additional equipment of NK-92 cells with a GD2-specific CAR leads to a significant lysis of NB cells in vitro and in vivo [20]. Furthermore, the additional inhibition of TIGIT in NK-92 cells leads to improved cytotoxicity against acute myeloid leukaemia in recent studies [21, 22].

This study aimed to further evaluate the effect of the PVR/PVRL2-TIGIT in NB and analyse the combinatory effect of GD2-CAR NK-92 with CRISPR-based TIGIT knockout in NB treatment.

## Materials and methods

### RNA-seq data analysis

We used previously published RNA-seq data from Harenza et al. [23] to assess the expression of the surface molecules. PD-L1, B7-H3, PVR, PVRL2, HVEM and B7-H4 were chosen for analysis via FPKM (fragments per kilobase million) for five different NB cell lines: SK-N-AS, SH-SY5Y, IMR-32, Kelly and SK-N-SH. The heatmap was generated using R-based hierarchical clustering (ward.D2).

The Kaplan–Meier survival analysis was conducted using the *R*^2^: Genomics Analysis and Visualization Platform (http://r2.amc.nl) with default settings. The event-free and overall survival rates were analysed using the SEQC dataset (GSE49710) containing mRNA expression data from 498 primary NB samples [24]. For cutoff determination, scan modus with a minimal group number of eight was used generating two groups and performing a log-rank test. The best *p*-value and corresponding cutoff were used after the inclusion of a correction from multiple testing (Bonferroni-corrected *p*-values).

### Cell lines and cell culture conditions

The following human NB cell lines were used for this study: SH-EP, SK-N-LO, LAN-1, LS, SH-SY5Y, SK-N-AS, and Kelly. The cells were cultured at 37 °C and 5% CO_2_ in RPMI 1640 medium with 10% FBS heat-inactivated (h.i.), 10 mM HEPES, 100 U/ml penicillin, 100 µg/ml streptomycin and 0.5 mM sodium pyruvate (all from Thermo Fisher Scientific). NK-92 (ATCC, CRL-2407) were cultured using alpha MEM medium (Merck) with 12.5% FBS h.i. (Thermo Fisher Scientific), 12.5% horse serum h.i. (Sigma-Aldrich), 1% penicillin/streptomycin and 100 U/ml IL-2 (Peprotech). K562 (DSMZ, ACC 355), were cultured using RPMI 1640, 10% FBS h.i., and 1% penicillin/streptomycin. HEK 293 T (DSMZ, ACC 635) were cultured using DMEM medium (Thermo Fisher Scientific) with 10% FBS h.i. and 1% penicillin/streptomycin.

PBMCs from six healthy donors were isolated after whole blood after lysis of red blood cells using BD FACS™ Lysing solution (BD Biosciences). Cells were directly used for flow cytometry or cultured using X-Vivo® 15 medium (Lonza) with 10% FBS h.i., 1% penicillin/streptomycin, and 2 mM L-glutamine (Thermo Fisher Scientific). To assess if stimulation with interleukins changes the expression of IC, isolated PBMCs were cultured for 24 h at four different conditions: without additives, with 100 U/ml IL-2 (Peprotech), with 20 ng/ml IL-15 (Miltenyi Biotech) and with IL-2 + IL-15. To differentiate NK cells, staining with anti-CD56 and anti-CD3 was performed.

### Flow cytometry

Expression levels of ICs and GD2 were assessed by flow cytometry on a BD LSRFortessa Cell Analyzer (BD Biosciences) and a MACSQuant® Analyzer 10 (Miltenyi Biotec). Staining with antibodies (Supplementary material, Table 1) was performed using 1*10^6^ cells in FACS buffer. Data were analysed using the FlowJo Software (FlowJo LLC) and if necessary, the geometric mean fluorescent intensity (gMFI) ratio was calculated by dividing the MFI sample value by the MFI isotype control value.

### Generation of knockout cell lines using CRISPR/Cas9

The PVR, PVRL2, and TIGIT single and double knockout cell lines were generated using the CRISPR/Cas9 system. Guide RNAs were designed using the CCTop software [25]. The sequences (Supplementary material, Table 2) were cloned into lentiviral CRISPR vector constructs (pL40C-CRISPR.EFS.cerulean or pL40C-CRISPR.EFS.dTomato) [26]. Target cell lines were transduced with concentrated vsv-g pseudotyped lentiviral particles [27] and selected for receptor-negative cells using a BD FACSAria (BD Bioscience) or MACSQuant® Tyto® (Miltenyi Biotec).

### Generation of GD2-CAR NK-92 cells

Heavy and light chain sequences of a monoclonal antibody targeting GD2 (ch14.18) were amplified from pDHL7_ch14.18, connected using a (G_4_S)_3_ linker and cloned into pBullet-scFv_IRES_GFP [28]. Gamma-retroviral particles were produced as previously described [29] using the GALV envelope [30]. NK-92 cells were transduced (MOI 15) with Vectofusin-1 (Miltenyi Biotec) according to the manual. The cells were sorted as a bulk sort for GFP-positive populations as described above.

Expression of the CAR was analysed by staining the variable light chains with protein L as previously described [31] after blocking Fc receptors using Human TruStain FcX™ (BioLegend). The sample was stained with 0.05 µg Streptavidin-APC/Cyanine 7 (BioLegend) at RT for 20 min and analysed by flow cytometry.

### Flow cytometry-based cytotoxicity assay

After staining the target cells with 5 µM eFluor™ 670 (eBioscience™) according to the manufacturer’s instructions, they were resuspended with cytotoxicity medium (RPMI medium, 2% FBS h.i., 1% L-Glutamine, 1% penicillin/streptomycin, 0.5 mM sodium pyruvate). 4*10^5^ cells/ml target cells were seeded into 96-well round-bottom plates. 4*10^5^ cells/ml (ratio 1:1) or 2*10^6^ cells/ml (ratio 5:1) NK-92 cells were added. For maximal lysis, 1% Triton X-100 (Carl Roth) was added. For spontaneous lysis, cytotoxicity medium only was added. The plate was centrifuged at 100 × g for one minute and incubated at 37 °C for four hours. After co-culture, cells were stained with propidium iodide solution (BioLegend) and analysed by flow cytometry. The specific lysis was calculated using the following formula: specific lysis [%]  =  (lysis – spontaneous lysis) / (maximal lysis – spontaneous lysis) *100.

### Incucyte®-based cytotoxicity assay

The cell lines SK-N-LO and LAN-1 were labelled using the Incucyte® Nuclight Red Lentivirus reagent (Sartorius AG). A stable cell line was generated by puromycin selection and cell sorting as described above. For the assay, 1.5*10^4^ NB cells were seeded into a 96-well plate and incubated overnight. On day two, NK-92 cells were added at effector-target ratios 1:1 and 5:1. Medium was replaced by cytotoxicity medium (see above) and placed in the Incucyte® SX5 Live Cell Imaging System (Sartorius AG) chamber. Measurement was performed at 0, 2 and every 4 h for 24 h using cell-by-cell analysis settings. Three images per well were taken. For analysis, the number of red cells equivalent to the number of viable cells was calculated and normalised to 0 h.

### Degranulation assay

The target cells were stained with eFluor™ 670 (eBioscience™) as described above. 1*10^5^ NK-92 cells were seeded in 96-well round bottom plates. Target cells were added at an effector target ratio of 1:1. Maximal degranulation was determined by adding 2.5 µg/ml PMA and 5 µg/ml ionomycin. Background degranulation was assessed using medium only. An anti-CD107a antibody was added before incubation. After 4 h of co-culture, flow cytometry was performed. NK-92 cells were gated as eFluor negative and BV421™-CD107a positive cells.

### Cytokine secretion

1*10^5^ cells/ml effector and target cells (ratio 1:1) were seeded into 96-well round-bottom plates. For maximum interferon-γ production, 40 ng/ml PMA and 4 ng/ml Ionomycin were added to effector cells. The co-cultures were incubated at 37 °C and 5% CO_2_ for 18 h. 100 µl of the supernatant was used to quantify interferon-γ production using the INFgamma Human ELISA Kit with a Varioskan™ LUX multimode microplate reader (both Thermo Fisher Scientific). Normalisation of the results to the interferon-γ release without any activation was performed to eliminate effects such as different vitalities of the NK-92 cells.

### Statistical analysis

Statistical analysis was performed using GraphPad Prism 5.0. Data were assessed for similarity of variance and statistical analysis was performed by 2way ANOVA or ordinary one-way ANOVA (Dunnett’s multiple comparison test, Sidak’s multiple comparison test, Brown-Forsythe test, and Bartlett’s test). Horizontal bars represent means ± standard deviation. *p* < 0.05, *p* < 0.01, *p* < 0.001 and *p* < 0.0001 were considered statistically significant and are indicated by *, **, *** or **** respectively.

## Results

### PVR and PVRL2 as potential IC molecules

**Fig. 1:**
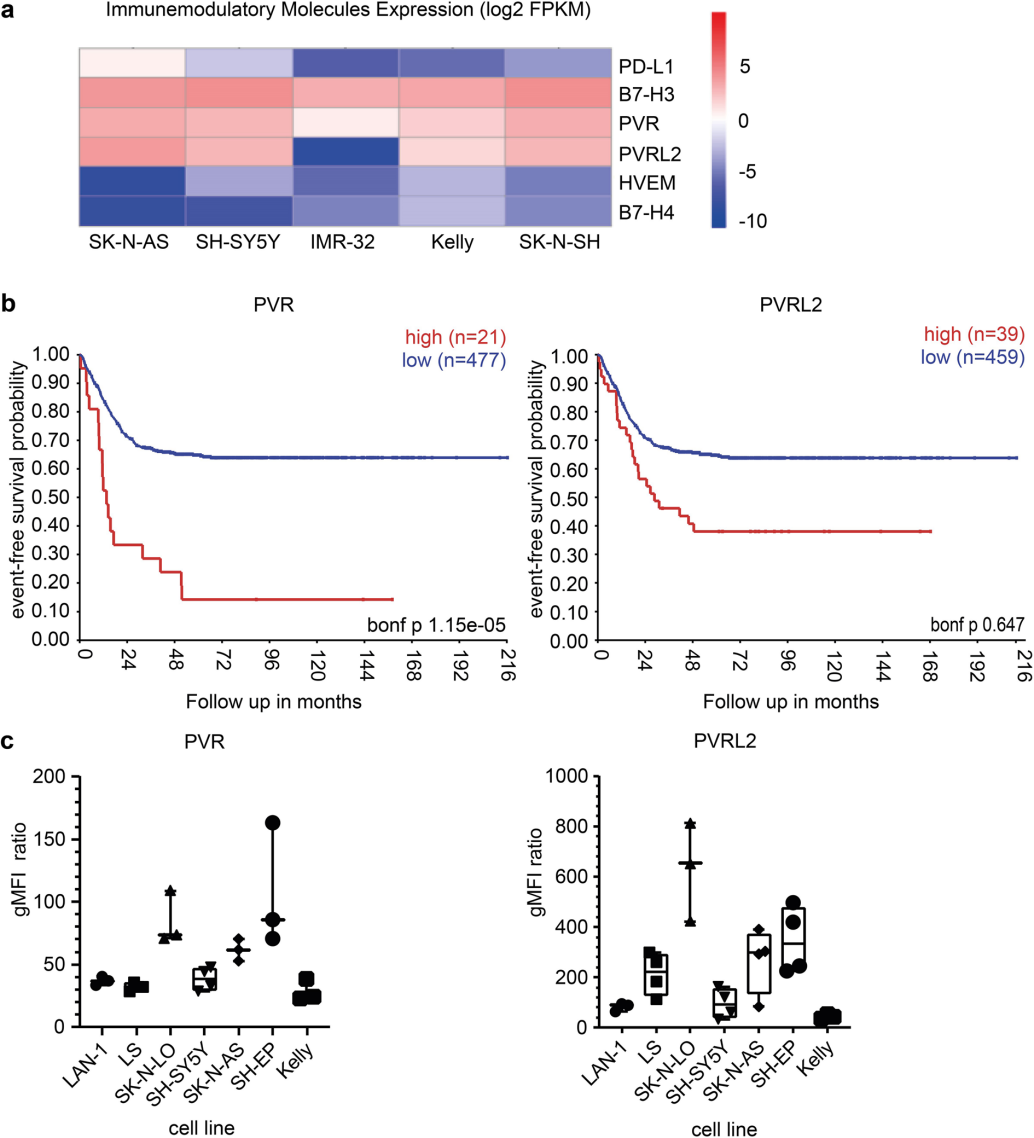
**Identification of PVR and PVRL2 as potential immune checkpoint molecules.** (**a**) Heatmap analysis depicting genes for immunomodulatory molecules. Gene set was manually curated. Bars are color-coded according to the expression value FPKM as indicated in the expression scale. Columns represent different NB cell lines: SK-N-AS, SH-SY5Y, IMR-32, Kelly and SK-N-SH. Data were mean-centred, and rows were clustered using ward.D2 clustering method. RNA-Seq Data by Harenza et al. [23]. (**b**) Kaplan–Meier-Curves based on the expression of PVR and PVRL2 in primary NB samples. The curves were generated with http://r2.amc.nl and RNA-Seq data by Zhang et al. [24]. Event-free survival probability with high or low expression of PVR and PVRL2, all tumour stages. (**c**) Surface expression of PVR and PVRL2 on NB cell lines LAN-1, LS, SK-N-LO, SH-SY5Y, SK-N-AS, SH-EP and Kelly. The surface expression was analysed via flow cytometry (*n* = 3). Shown is the mean fluorescent intensity (MFI) ratio

Using previously published RNA sequencing data by Harenza et al. [23] from the NB cells lines SK-N-AS, SH-SY5Y, IMR-32, Kelly, and SK-N-SH, we analysed the expression of IC molecules (Fig. 1a). The data showed an enhanced expression of PVR, PVRL2, and B7-H3 on the NB cell lines compared to the expression of PD-L1, HVEM and B7-H4.

After identifying the transcription profile in cell lines, we analysed published RNA-Seq data of NB patient samples by Zhang et al. (Fig. 1b) [24]. Through Kaplan–Meier curves plotting the event-free survival of patients with high and low PVR and PVRL2 expression (MYCN ± , all stages, all risk groups), we demonstrated that high PVR correlates significantly with lower event-free survival probability and vice versa (Fig. 1b). Although high PVRL2 expression was associated with a lower event-free survival, this correlation did not reach statistical significance. In addition, high PVR expression resulted in a lower overall survival probability. However, high PVRL2 expression was associated with a higher overall survival probability (Fig. S1a). For patients with high-risk tumours, a high expression of PVR also led to a reduced event-free and overall survival whereas a high expression of PVRL2 led only to a lower event-free survival probability (Fig. S1b).

To validate the RNA-Seq data for NB cell lines, we analysed the protein expression of PVR and PVRL2 on the surface of the following cell lines by flow cytometry: LAN-1, LS, SK-N-LO, SH-SY5Y, SK-N-AS, SH-EP and Kelly (Fig. 1c). PVR was highly expressed on SK-N-LO, SH-EP, and SK-N-AS, while LAN-1, LS, SH-SY5Y, and Kelly showed low expression. PVRL2 was highly expressed on all cell lines assessed, particularly on LS, SK-N-LO, SK-N-AS, and SH-EP, but to a lesser extent on LAN-1, SH-SY5Y, and Kelly cells. Based on these observations, we selected the following cell lines for our experiments: LAN-1 (PVR^dim^PVRL2^dim^), SK-N-LO (PVR^bright^PVRL2^bright^), and SH-EP (PVR^bright^PVRL2^bright^). LAN-1 and SK-N-LO were also positive for the ganglioside GD2, but SH-EP were negative (Fig. S2).

### Efficient CRISPR/Cas9-based knockout of PVR and PVRL2 on NB cell lines

**Fig. 2:**
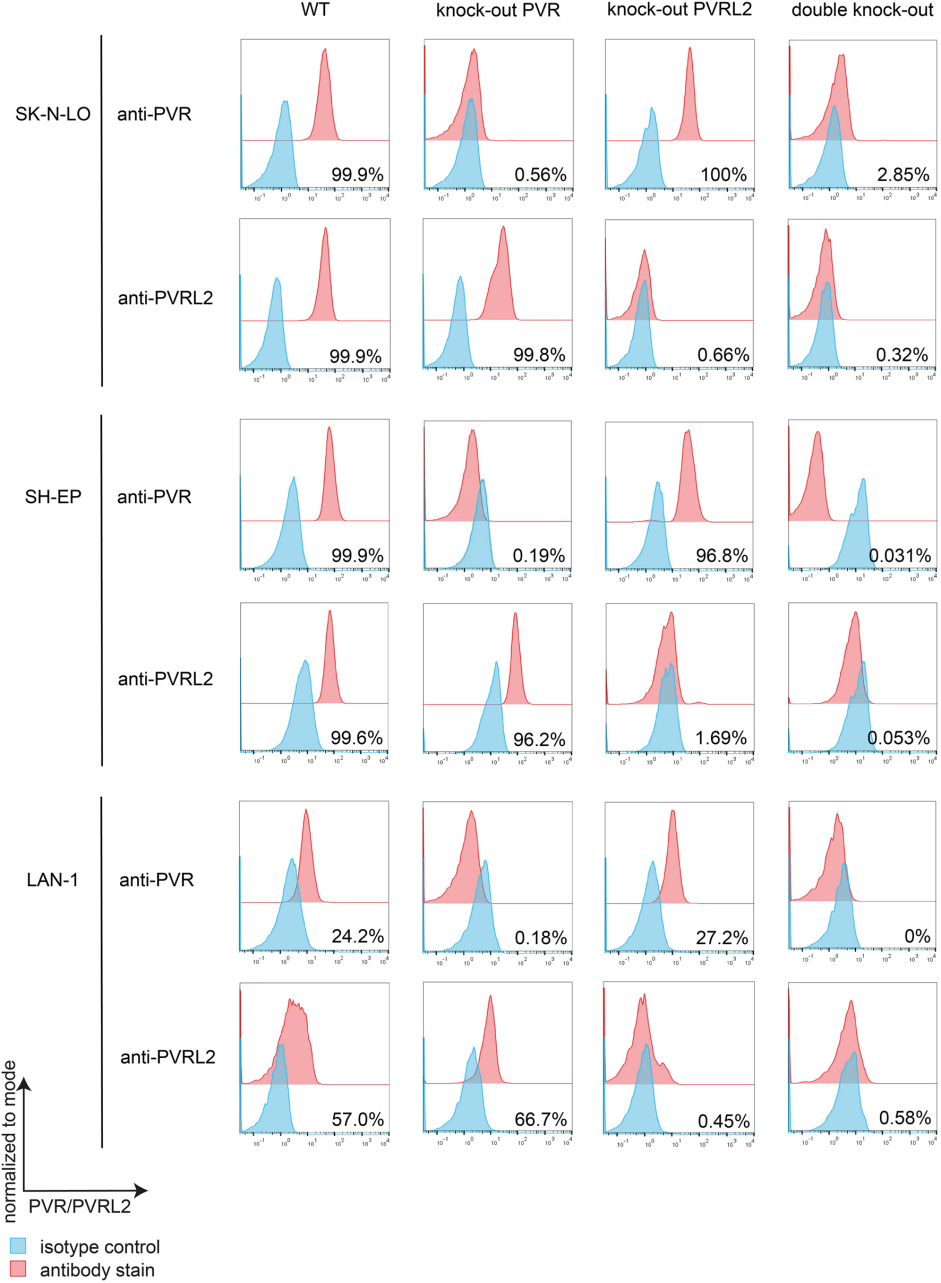
**Knockout of PVR and PVRL2 in cell lines.** Surface expression of PVR and PVRL2 wildtype and knockout cell lines SK-N-LO, SH-EP and LAN-1. The surface expression was analysed via flow cytometry (*n* = 3) after antibody staining and staining with the corresponding isotype control. Shown is one representative example

To directly compare receptor-bearing and receptor-free cells within a single cell line, we genetically modified the cell lines SK-N-LO, SH-EP, and LAN-1 using CRISPR/Cas9 and sorted for single-cell clones. Thereby we obtained both, single and double knockout clones. These clones were evaluated for receptor expression revealing that the selected single-cell clones from all cell lines showed a complete absence of the respective IC molecules on their surface (Fig. 2).

**Fig. 3:**
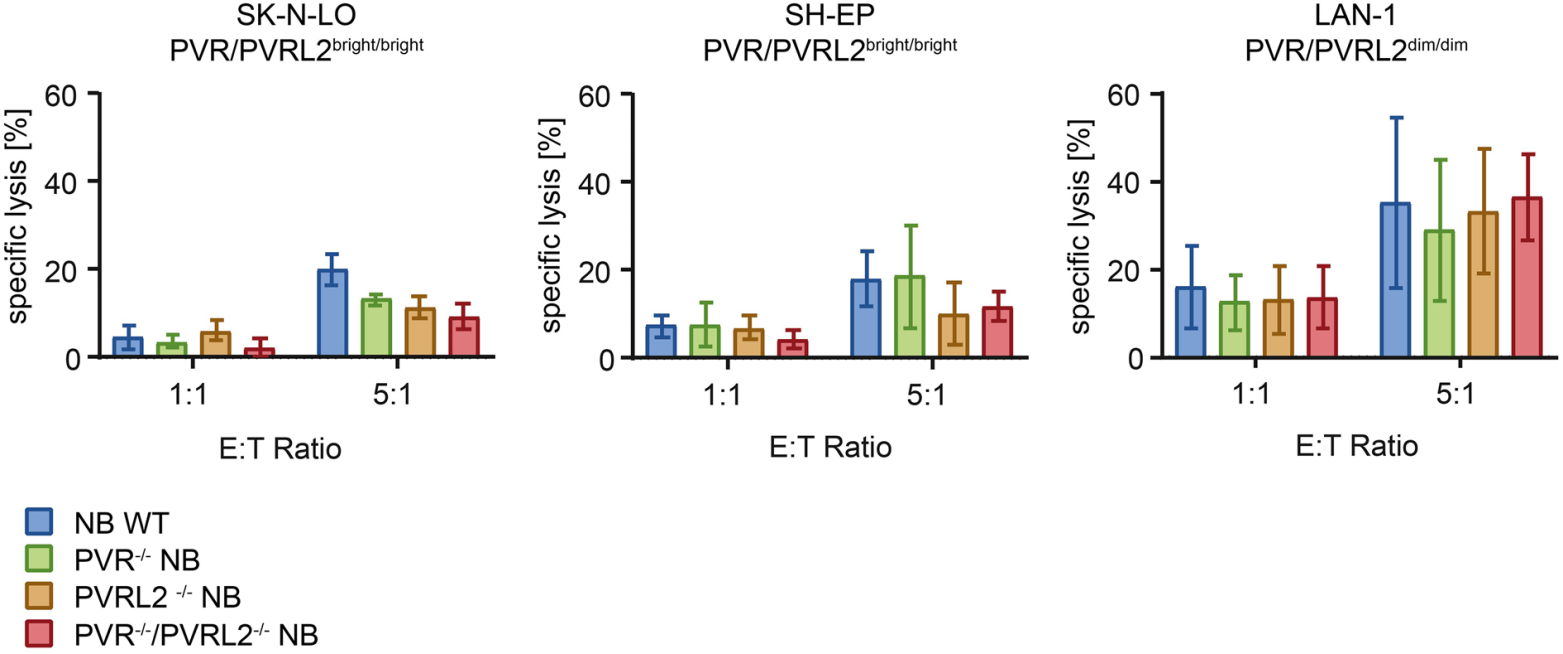
**Cytotoxicity of NK-92 cells against knockout NB cell lines.** Cytotoxicity assays showed no improved lysis of NB cells without PVR or PVRL2 expression on SK-N-LO, SH-EP and LAN-1 cells (*n* = 3). Error bars showing the standard deviation of triplicates of three assays. Specific lysis was calculated for each target cell line using Triton X-100 as maximal lysis and addition of medium as spontaneous lysis

To evaluate the effect of the missing inhibitory receptors PVR and PVRL2 on NB cells towards NK-92 cell-mediated killing, we performed cytotoxicity assays (*n* = 3). These experiments did not show enhanced lysis of NB cells with or without the receptors PVR or PVRL2 (Fig. 3).

To address if other TIGIT-interacting receptors are present on NB cells, we assessed the expression of Nectin-3 and Nectin-4. Nectin-4 was not expressed on any of the three NB cell lines, whereas all wildtype NB cells were positive for Nectin-3. In the SH-EP and LAN-1, the expression did not change after knock-out of PVR, PVRL2 or both. However, in the SK-N-LO cells the knockout of PVR or PVRL2 lead to the loss of Nectin-3 on the surfaces of the cells (Fig. S3).

### Expression of PVR and PVRL2 interaction partners on NK-92 cells

**Fig. 4:**
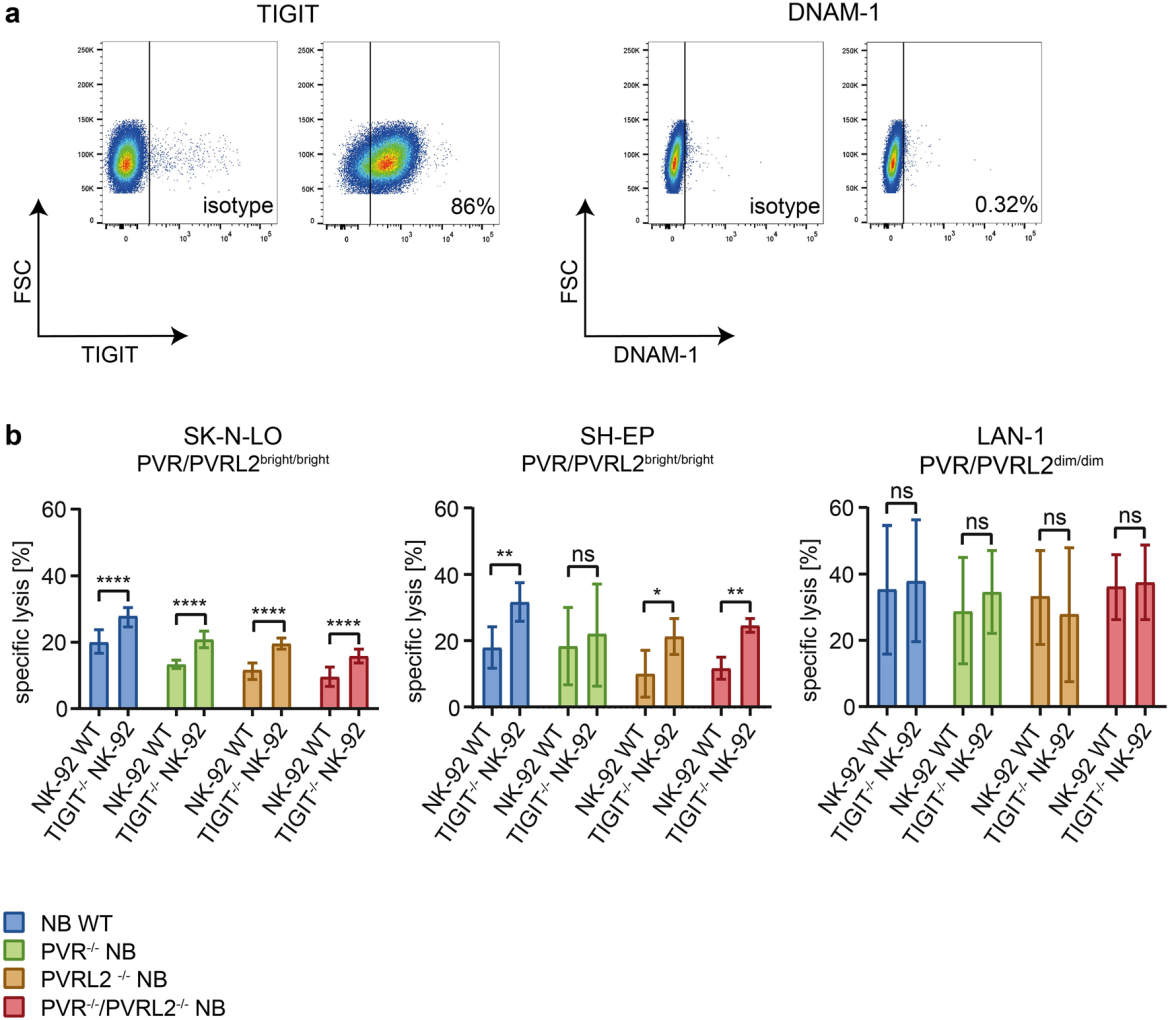
**Identification and function of PVR/PVRL2– interaction partners on NK-92 effector cells.** (**a**) Surface expression of immune checkpoint molecules on NK-92 cells. The surface expression was analysed via flow cytometry (*n* = 3). Shown is one representative example. (**b**) Cytotoxicity assays of NK-92 cells compared to NB knockout cells in a 5:1 ratio (*n* = 3). Error bars showing the standard deviation of triplicates of three assays. *p* < 0.05, *p* < 0.01, *p* < 0.001 or *p* < 0.0001 were indicated by *, **, *** or **** respectively

Subsequently, we evaluated the surface expression of IC molecules on NK-92 and pNK cells from healthy donors by flow cytometry. The NK-92 cell line demonstrated no expression of the activating receptor DNAM-1 (0.32%) and a high expression of the inhibitory receptor TIGIT (86%) (Fig. 4a). PD-1 (3.06%) and CTLA-4 (1.93%) were expressed only at a very low frequency (Figs. S4a and S4b). However, on pNK cells, the inhibitory receptor TIGIT was not expressed (0.39%) and the activating receptor DNAM-1 (99.2%) was strongly expressed. pNK cells expressed PD-1 (9.23%) and not CTLA-4 (0.0009%).

To analyse whether stimulation of pNK cells with IL-2 and IL-15 alters the expression of the receptors, we performed short-term cultures of cells with IL-2, IL-15, or a combination of both. After 24 h there was no significant change in receptor expression (Fig. S4c). We also detected no differences between the stimulation with IL-2 or IL-15.

### Knockout of TIGIT on NK-92 cells led to enhanced cytotoxicity against NB cell lines

We aimed to analyse the inhibitory interactions on the effector cell side by targeting the PVR/PVRL2 binding receptor TIGIT. We used the CRISPR/Cas9 gene-editing technique to modify the expression of TIGIT in NK-92 cells. After successfully transducing and sorting the TIGIT-negative population of NK-92 cells, the analysis revealed a significant reduction in TIGIT expression and successful generation of TIGIT^−/−^ NK-92 cells (Fig. S5a).

To assess the impact of this modification, we compared co-cultures of wildtype NB cell lines with wildtype NK-92 cells or TIGIT^−/−^ NK-92 cells (Fig. 4b). Compared to wildtype NK-92, the TIGIT^−/−^ NK-92 cells showed an increased target cell lysis of SK-N-LO (PVR/PVRL2^bright/bright^) and SH-EP (PVR/PVRL2^bright/bright^) cells. Against LAN-1 (PVR/PVRL2^dim/dim^) cells, the knockout of TIGIT did not alter the NK-92 cell-mediated killing. Furthermore, we co-cultured NB knockout cells with wildtype NK-92 cells and TIGIT^−/−^ NK-92 cells. These experiments assessed if disrupting inhibitory signals on both sides could additionally enhance the overall effect. As a result, TIGIT^−/−^ NK-92 or wildtype NK-92 cells did not show an enhanced cytotoxicity when cultured with knockout target cells compared to wildtype NB cells (Fig. 4b). But we did observe an enhanced lysis by TIGIT^−/−^ NK-92 against PVR^−/−^ SK-N-LO, PVRL2^−/−^ SK-N-LO, PVR^−/−^/PVRL2^−/−^ SK-N-LO, PVRL2^−/−^ SH-EP and PVR^−/−^/PVRL2^−/−^ SH-EP cells. The lysis of LAN-1 could not be enhanced by the knockout of TIGIT on NK-92 cells.

### Additional activating signals improved cytotoxic activity

The effector cell line NK-92 demonstrated no expression of the activating ligand DNAM-1 (Fig. 4a). This led us to the assumption that the overall activation potential of these cells was reduced. To improve their cytotoxic activity against NB cells, we equipped the NK-92 with a GD2-CAR construct already used in clinical trials with CAR T cells [7, 32]. We hypothesized that a combination of the blockade of the inhibitory signal by TIGIT knockout and specific activation of NK-92 cells by a CAR could lead to enhanced lysis. TIGIT^−/−^ NK-92 cells were transduced with a GD2-CAR-bearing vector (Fig. S5b) and subsequently sorted as a bulk sort to include multiple cell clones (Fig. S5c). As a control, GD2-CAR NK-92 (wildtype) cells were generated.

**Fig. 5:**
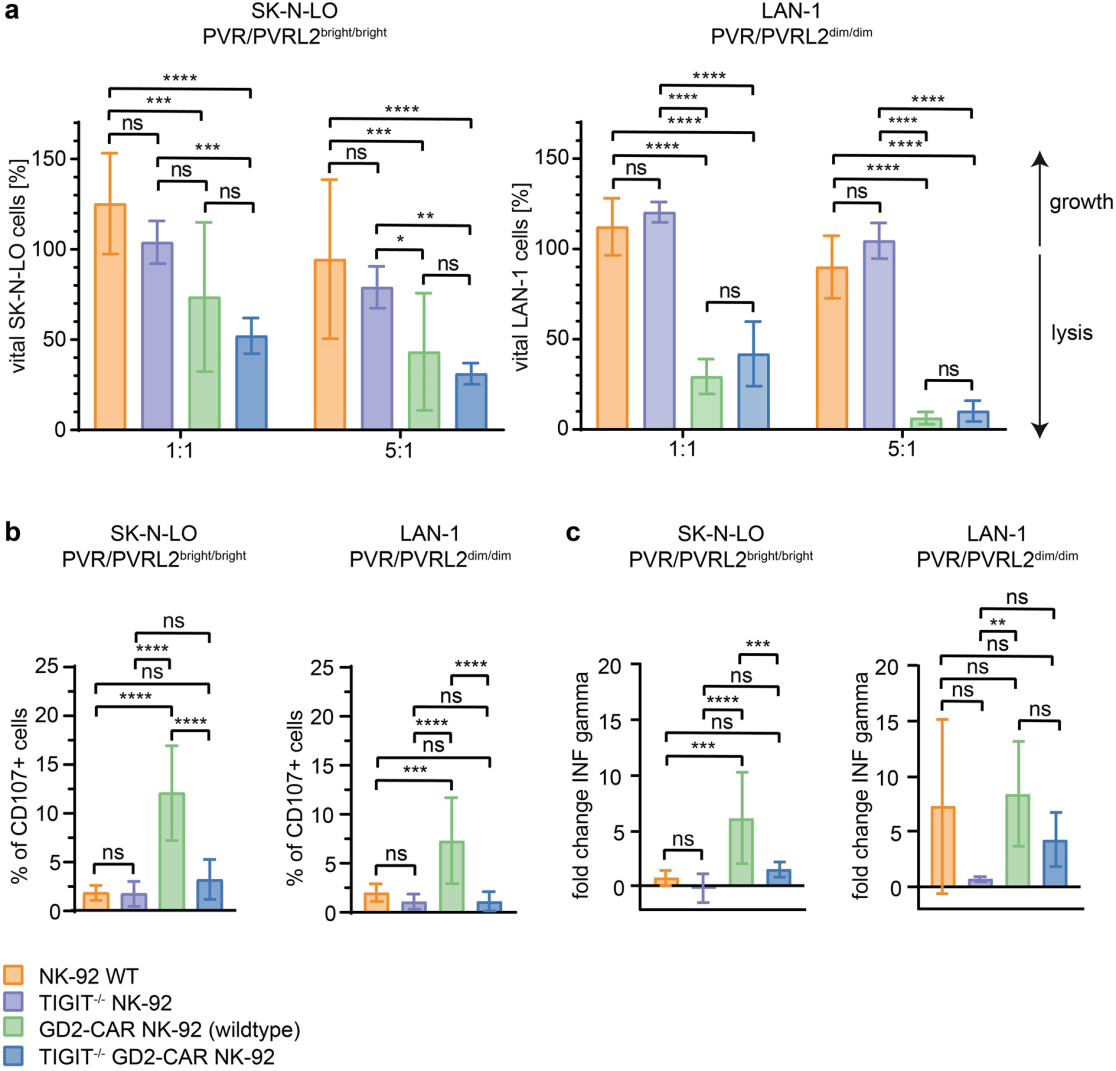
**Cytotoxicity of TIGIT**^**–/–**^ **GD2-CAR NK-92 cells.** (**a**) Cytotoxicity assays of NK-92 WT, TIGIT^-/-^ NK-92, GD2-CAR NK-92 (wildtype) and TIGIT^-/-^ GD2-CAR NK-92 cells against NB cell lines (*n* = 3) using the Incucyte® SX5 Live Cell Imaging System, results normalised to 0 h. Error bars showing the standard deviation of triplicates of three assays. *p* < 0.05, *p* < 0.01, *p* < 0.001 or *p *< 0.0001 were indicated by *, **, *** or **** respectively. (**b**) Degranulation assays of WT and modified NK-92 cells co-cultured with NB cell lines (*n* = 3), effector-target-ratio 1:1. Error bars showing the standard deviation of triplicates of three assays. *p* < 0.05, *p* < 0.01, *p* < 0.001 or *p* < 0.0001 were indicated by *, **, *** or **** respectively. (**c**) Interferon-γ release assays of NK-92 cells co-cultured with NB cell lines (*n* = 3). Results were normalized using fold change to interferon release without stimulation for each effector cell line. Error bars showing the standard deviation of triplicates of three assays. *p* < 0.05, *p* < 0.01, *p* < 0.001 or *p* < 0.0001 were indicated by *, **, *** or **** respectively

Consequently, we focussed on the cell lines with high GD2 expression: SK-N-LO and LAN-1 (Fig. S2). As shown in Fig. 5a, we confirmed the expected significantly enhanced lysis of both NB cell lines by the GD2-CAR NK-92 cells in comparison to wildtype NK-92. Surprisingly, the TIGIT^−/−^ GD2-CAR NK-92 cells did not show a significantly enhanced cytotoxicity against SK-N-LO or LAN-1 cells compared to the GD2-CAR NK-92 cells.

To analyse whether the previous results are based on an enhanced activation of NK-92, we performed degranulation and interferon-γ release assays (*n* = 3). As controls, PMA/ionomycin stimulation and K562 cells were used to compare activation levels of all NK-92 subtypes in the degranulation assays (Fig. S6). After co-culture with SK-N-LO or LAN-1 cells, only the GD2-CAR NK-92 cells showed an increased degranulation (Fig. 5b). In the interferon-γ release assay, we detected an enhanced secretion of GD2-CAR NK-92 cells after co-culture with the SK-N-LO. But as shown before, TIGIT^−/−^ GD2-CAR NK-92 cells did not show higher interferon-γ levels in our assay (Fig. 5c). These results supported the previous experiments and proved that a combination of a TIGIT knockout and a GD2-CAR does not enhance the reaction of NK-92 cells towards NB cells.

## Discussion

Our results demonstrate that similar to other cancer entities [13], the PVR/PVRL2-TIGIT checkpoint axis could serve as a new immunotherapeutic target for NB. Based on publicly available RNA-Seq data [24, 33], we identified the expression of PVR and PVRL2 in NB primary samples and cell lines (Fig. 1). We showed that a high expression correlated with lower survival probability of patients. Since PVR is particularly expressed in lower-stage NB [34] and PVRL2 is associated with advanced tumour stage, rapid progression, and high malignancy [35], both receptors are worth consideration in the evaluation of further therapeutical approaches.

Since the elimination of PVR or PVRL2 in NB cell lines was not sufficient to generate a higher cytotoxic activity of NK-92 cells in vitro (Fig. 3)*,* we presume that a modification of the tumour cells alone cannot sufficiently improve the lysis. As described before, this checkpoint axis is a sophisticated system of different receptors, each with different effects on the immune response [17, 36]. However, previous results suggested that the interaction of TIGIT and PVR had a greater impact on cytotoxicity than other inhibiting receptors [37]. On the used NB cells, we could also detect Nectin-3 (Fig. S3), which also binds to TIGIT [38]. The presence of this receptor on most of the cell lines could explain why the knockout of PVR and PVRL2 did not lead to a significantly increased lysis. The loss of Nectin-3 in the SK-N-LO cells with PVR/PVRL2-KO could be explained by a necessary interaction of PVRL2 and Nectin-3, which on the other hand does not explain the unaffected lytic efficiency. Therefore, even more interactions might be present, which need more analyses.

To target this IC axis, it is also important to assess the receptor profile on the effector cells. We detected a significant difference between the expression of TIGIT or DNAM-1 on pNK cells from healthy donors and NK-92 cells that did not change after stimulation of pNK with IL-2/IL-15 (Figs. 4 and S4). The IC expression on NK-92 cells is comparable to the previously reported IC expression profile on tumour-infiltrating NK cells in other cancer entities with a DNAM-1 down-regulation and an upregulation of TIGIT [39, 40]. Recent results suggest similar expression profiles for tumour-infiltrating NK cells in NB [41]. The low expression of DNAM-1 on tumour-infiltrating NK cells should be considered carefully since modulating the inhibitory part of the axis can only be effective if the activating ligands (DNAM-1) are highly expressed and the activating part of the axis is functional.

During our studies, we modified the effector cell side by knocking-out TIGIT and could detect an enhanced lysis of SK-N-LO cells by TIGIT^−/−^ NK-92 cells (Fig. 4). Cytotoxicity against LAN-1 was unaltered as those cells express only low levels of PVR and PVRL2. Those results were consistent with other studies showing that blocking TIGIT had a positive effect on the cytotoxicity of NK-92 against other tumour entities [22]. Along these lines, it was shown that a combination of blocking antibodies against TIGIT and PD-L1 [16] or GD2 [15] led to improved survival of NB tumour-bearing mice.

Since the NK-92 cells did not express the activating ligand DNAM-1, we presumed that an activating signal could enhance the effect even further and introduced a GD2-CAR [42] into the TIGIT^−/−^ NK-92 cells (Fig. S5). Especially in solid tumours, CAR T cells suffer from exhaustion [43] and previous studies for B-ALL suggested that using a combination of TIGIT knockout with CD19-CAR NK cells improved their survival [21]. In our hands, the lytic effect of the TIGIT^−/−^ GD2-CAR NK-92 cells against SK-N-LO and LAN-1 was significantly improved compared to NK-92 wildtype cells, but the TIGIT knockout could not further improve the cytotoxicity of GD2-CAR NK-92 wildtype cells (Fig. 5). The lack of enhancement of GD2-CAR activity by a TIGIT knockout is likely caused by the presence of other receptors of this checkpoint network [36], which also influence the immune cell activity, or an exceeding signalling provoked by the GD2-CAR that is unaffected by other signalling pathways. We also did not observe an enhanced degranulation or interferon-γ release of TIGIT^−/−^ NK-92 cells which is comparable to previous studies [44] and could be explained by the absence of DNAM-1 on the NK-92 since experiments with SH-SY5Y cells demonstrated that DNAM-1 activation on NK-92 cells plays a crucial role in their degranulation activity [45].

Currently, there are TIGIT-directed blocking antibodies (e.g. tiragolumab, ociperlimab, vibostolimab) used in clinical trials [46]. So far, no trial with NB patients has been initiated. We propose that a combination of specific GD2-CAR cells, which are already being evaluated in trials [32] and immune checkpoint therapies may be an option, but other receptors of this checkpoint network must be evaluated as potential targets that could be utilized alone or in combination with TIGIT.

## Conclusion

Overall, this work confirmed that the PVR/PVRL2-TIGIT network plays a relevant role in NB. It underlines that this IC network consists of receptors, whose inhibition or activation alone is not sufficient for a therapeutic option. The deletion of TIGIT on NK-92 cells showed promising results, but further intensive research is needed to analyse this network in its entirety. A therapy targeting multiple receptors of this network or even beyond should be considered to successfully establish a checkpoint therapy in the treatment of NB that could be combined with GD2-CAR cells.

## Limitations

In our study, we had a look at PVR and PVRL2 on the tumour cell side and TIGIT and DNAM-1 on the immune effector cell side. As described, the PVR/PVRL2-TIGIT network consists of more receptors on both sides of the immunological synapse [36]. To further elucidate the best combination of immunotherapeutic approaches in NB, further experiments are necessary to identify the best combination of IC blockade for the treatment of NB, by targeting the whole network.

## Supplementary Information

Below is the link to the electronic supplementary material.

Supplementary file1 (PDF 1615 KB)

## Data Availability

The data that support the findings of this study are available on request from the corresponding author K.C.

## Abbreviations

B7-H3: B7 homolog 3, CD276
B7-H4: B7, homolog 4
CAR: Chimeric antigen receptor
CTLA-4: Cytotoxic T lymphocyte-associated protein 4, CD152
DNAM-1: DNAX accessory molecule-1, CD226
FPKM: Fragments per kilobase million
GD2: Ganglioside G2
gMFI: Geometric mean fluorescent intensity
HVEM: Herpesvirus entry mediator
IC: Immune checkpoint
ITIM: Immunoreceptor tyrosine-based inhibitory motif
NB: Neuroblastoma
PBMC: Peripheral blood mononuclear cell
PD-1: Programmed cell death protein 1, CD279
PD-L1: Programmed death-ligand 1
pNK: Primary NK cells
PVR: Poliovirus receptor, CD155
PVRL2: Poliovirus receptor-like 2, CD112
TIGIT: T cell immunoreceptor with Ig and ITIM domains
