# Supplementary material for: GD2-CAR NK-92 cell activity against neuroblastoma cells is insusceptible to TIGIT knockout
Source: Cancer Immunol Immunother. 2025 May 3;74(6):191. doi: 10.1007/s00262-025-04010-6 (PMC12049354; doi:10.1007/s00262-025-04010-6)
Supplement: Supplementary file 1 — Supplementary file1 (PDF 1615 KB) [file 262_2025_4010_MOESM1_ESM.pdf]

Supplementary Material

Tab. 1 List of antibodies used in this study

antigen	fluorophore	reactivity
PVR	APC/Fire™ 750	anti-human
PVRL2	APC	anti-human
Nectin 3	secondary AB	anti-human
Nectin 4	APC	anti-human
GD2	BV510™	anti-human
CD3	PE/Cyanine 7	anti-human
CD3	VioBlue®	anti-human
CD56	FITC	anti-human
DNAM-1	BV421™	anti-human
CTLA-4	APC	anti-human
PD-1	BV421™	anti-human
TIGIT	APC/Cyanine 7	anti-human
CD107a	BV421™	anti-human

secondary antibody	fluorophore	clone
goat anti-mouse IgG	APC	Poly4053

isotype control	fluorophore	clone
Mouse IgG1, κ Isotype Ctrl	APC/Fire™ 750	MOPC-21
Mouse IgG1, κ Isotype Ctrl	APC	MOPC-21
REA control, human IgG1	APC	REA293
Mouse IgG2a, κ Isotype Ctrl	BV510™	MOPC-173
Mouse IgG1, κ Isotype Ctrl	Pe/Cyanine7	MOPC-21
Mouse IgG1, k Isotype Ctrl	BV421™	MOPC-21
Mouse IgG2a, κ Isotype Ctrl	APC/Cyanine 7	MOPC-173

Tab. 2 List of guide RNAs

target gene	guide RNA
PVR	GATGTTCGGGTTGCGCGTAG
PVRL2	GATCGGCTGGGCGGTTTAGT
TIGIT	GTCGCTGACCGTGAACGATAC

Supplementary Figures

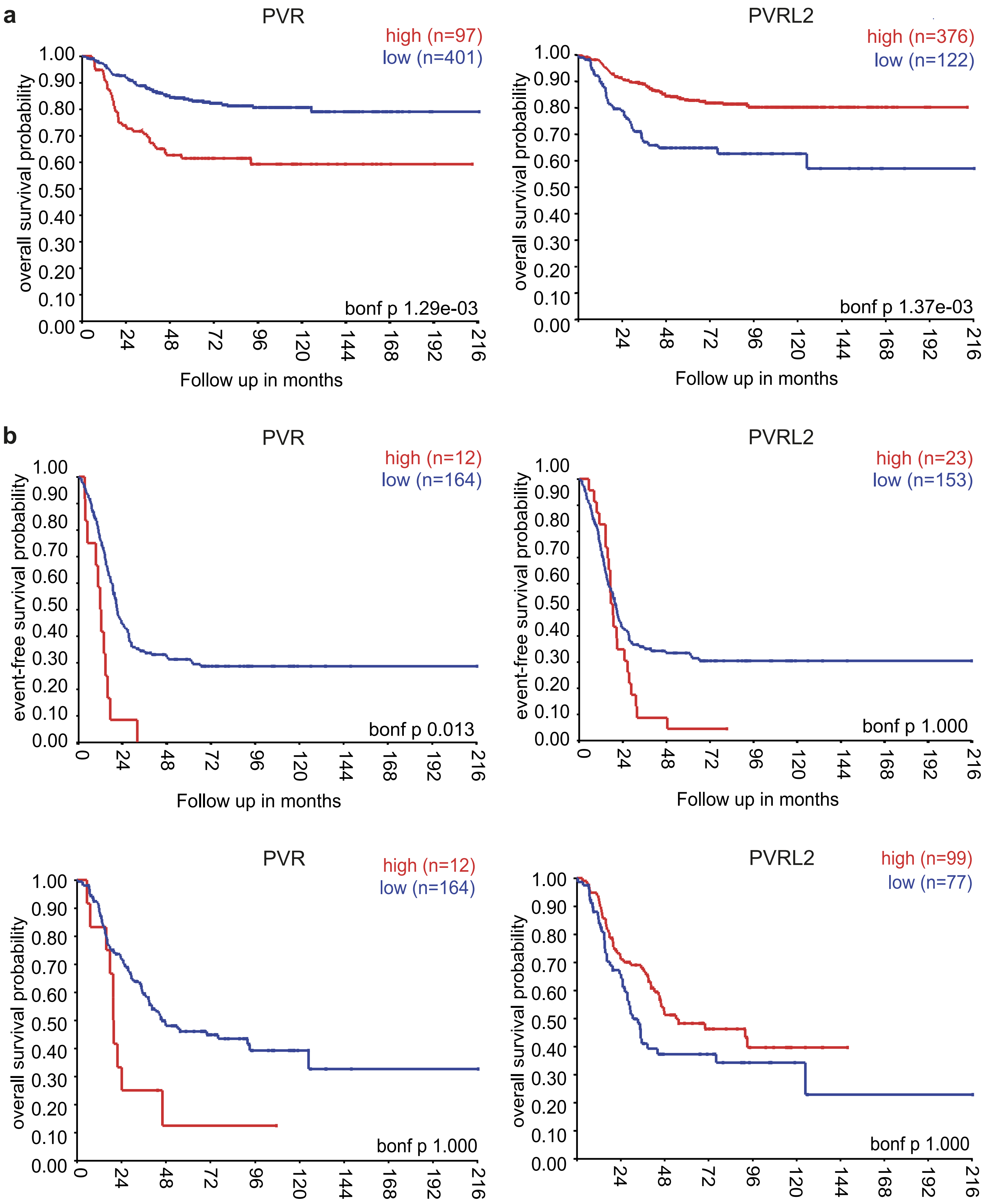

Fig. S1 Identification of PVR and PVRL2 as potential immune checkpoint molecules
Kaplan-Meier-Curves based on the expression of PVR and PVRL2 in primary NB samples. The curves were generated with http://r2.amc.nl and RNA Seq data was taken from Zhang et al., 2014.

a. Overall survival probability with high or low expression of PVR and PVRL2, all tumour stages.
b. Event-free survival probability and overall survival probability with high or low expression of PVR and PVRL2 in individuals with only high-risk tumours.

Supplementary Data

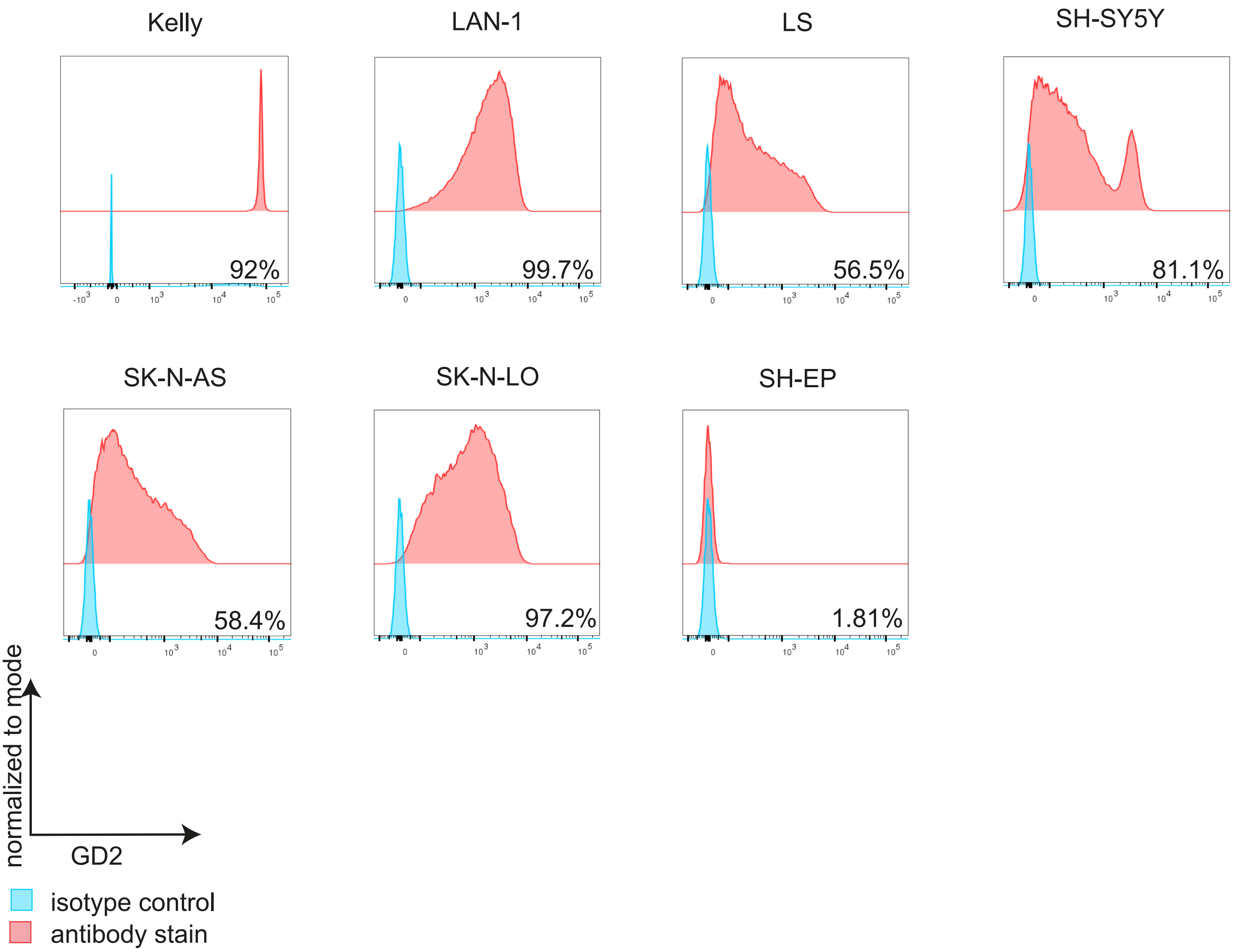

Fig. S2 Expression of GD2 on NB cell lines
Surface expression of GD2 on NB cell lines Kelly, LAN-1, LS, SH-SY5Y, SK-N-AS, SK-N-LO and SH-EP. The surface expression was analysed via flow cytometry.

Supplementary Data

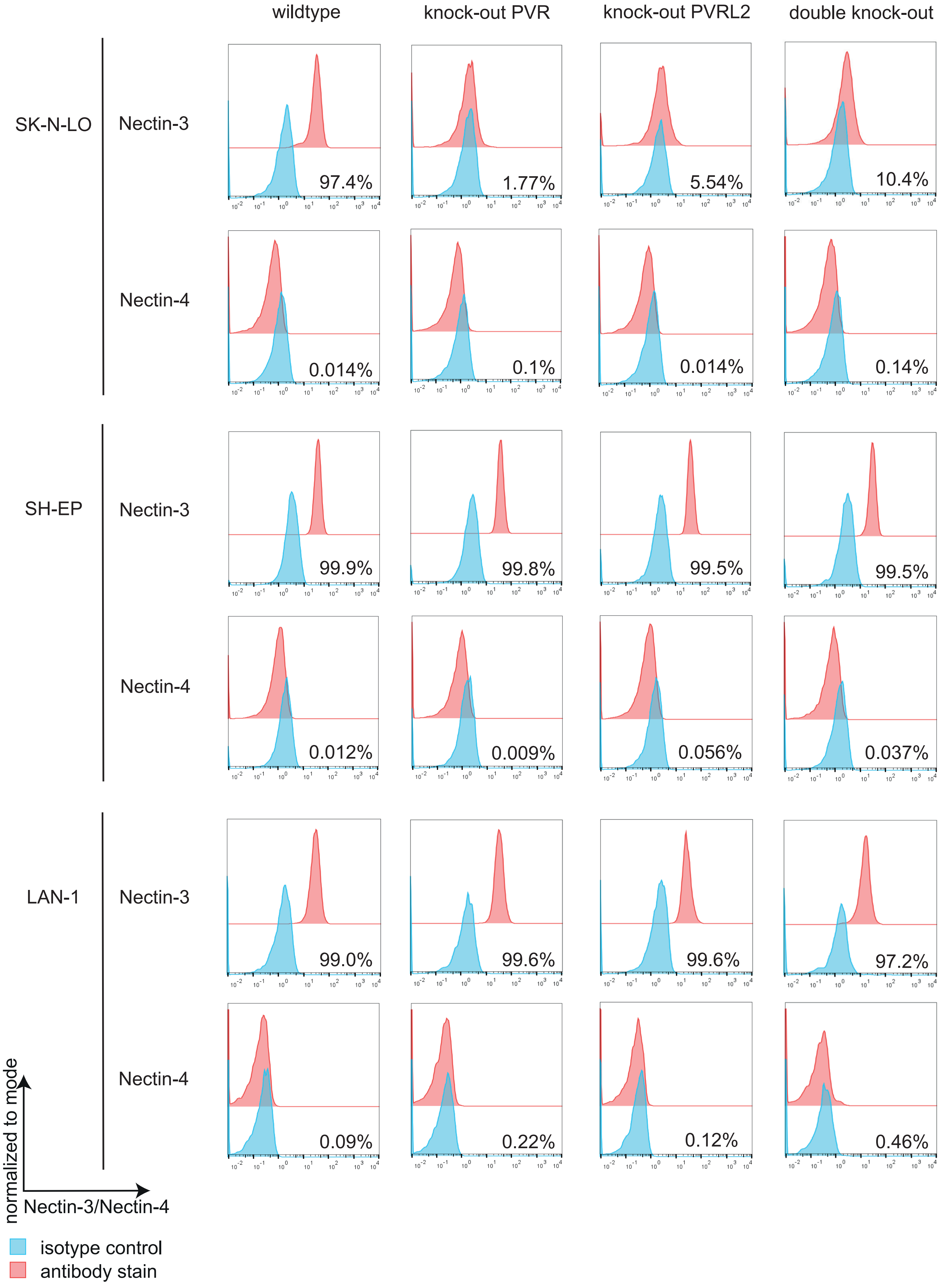

Fig. S3 Surface expression of TIGIT binding receptors Nectin-3 and Nectin-4
Surface expression of Nectin-3 and Nectin-4 on PVR and PVRL2 wildtype and knockout cell lines SK-N-LO, SH-EP and LAN-1. The surface expression was analysed via flow cytometry after antibody staining and staining with the corresponding isotype control. Shown is one representable sample from n=3 experiments.

Supplementary Data

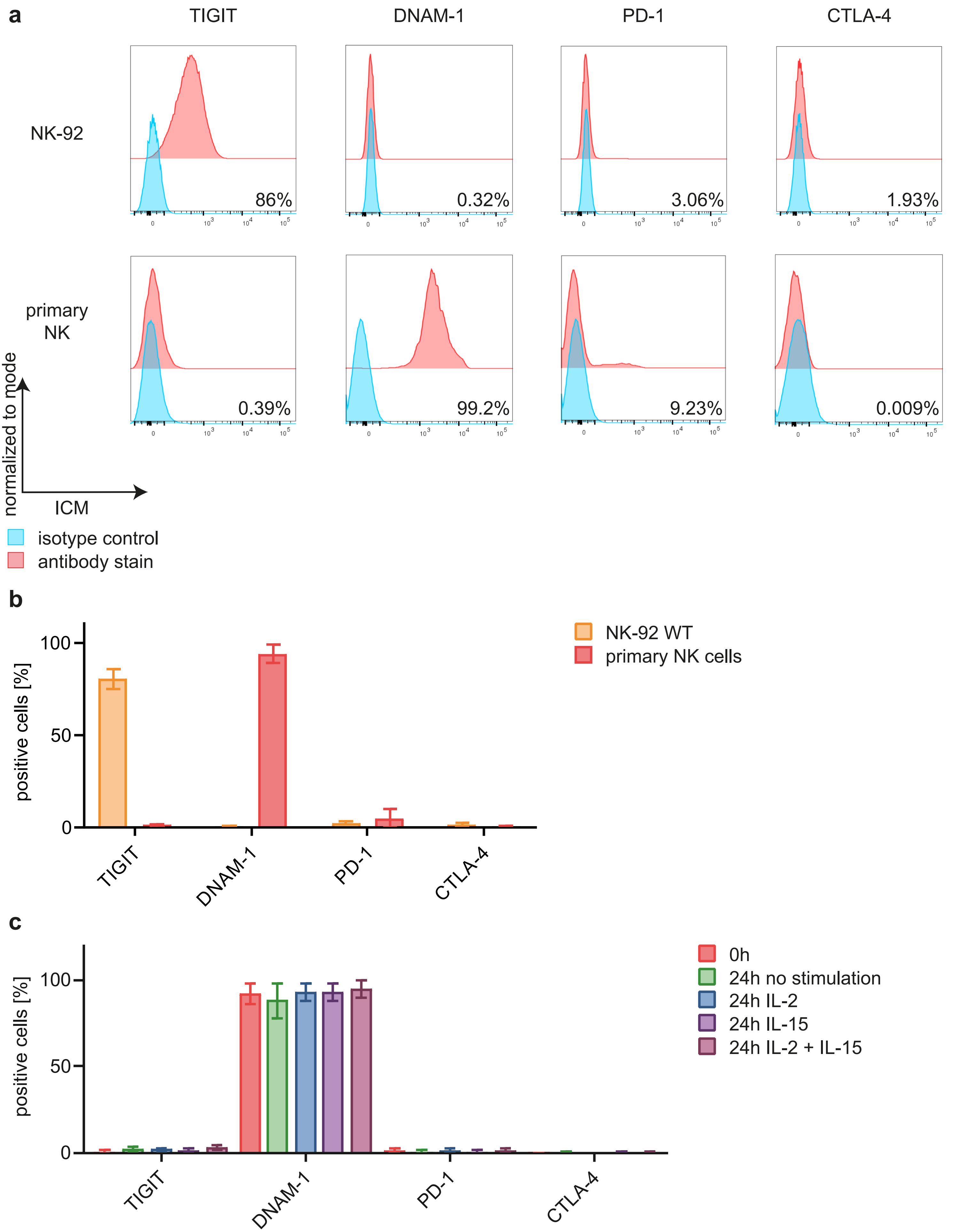

Fig. S4 Identification of PVR/PVRL2 – interaction partners on NK-92 effector cells

a. Surface expression of immune checkpoint molecules on primary NK cells and NK-92 cells analysed via flow cytometry. Shown is one representative sample.
b. Results from all experiments (NK-92 n=3, primary NK cells n=6).
c. Surface expression of IC molecules after stimulation of pNK cells with IL-2, IL-15 or IL2/IL-15 for 24 hours.

Supplementary Data

a

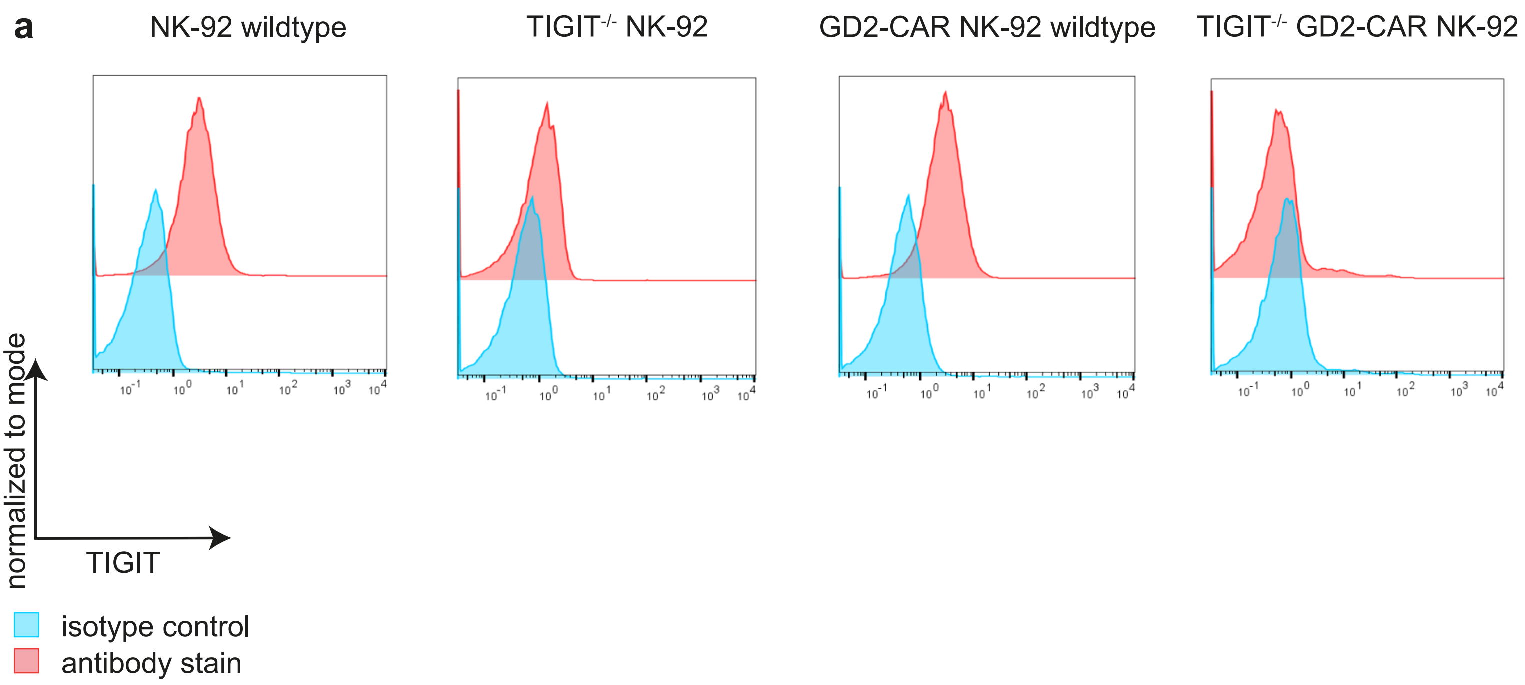

b

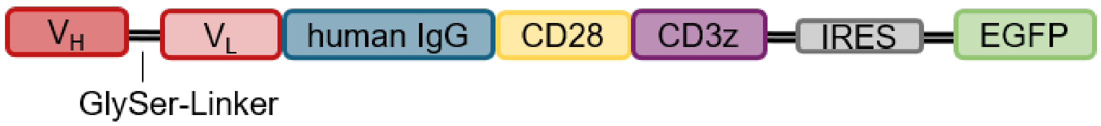

c

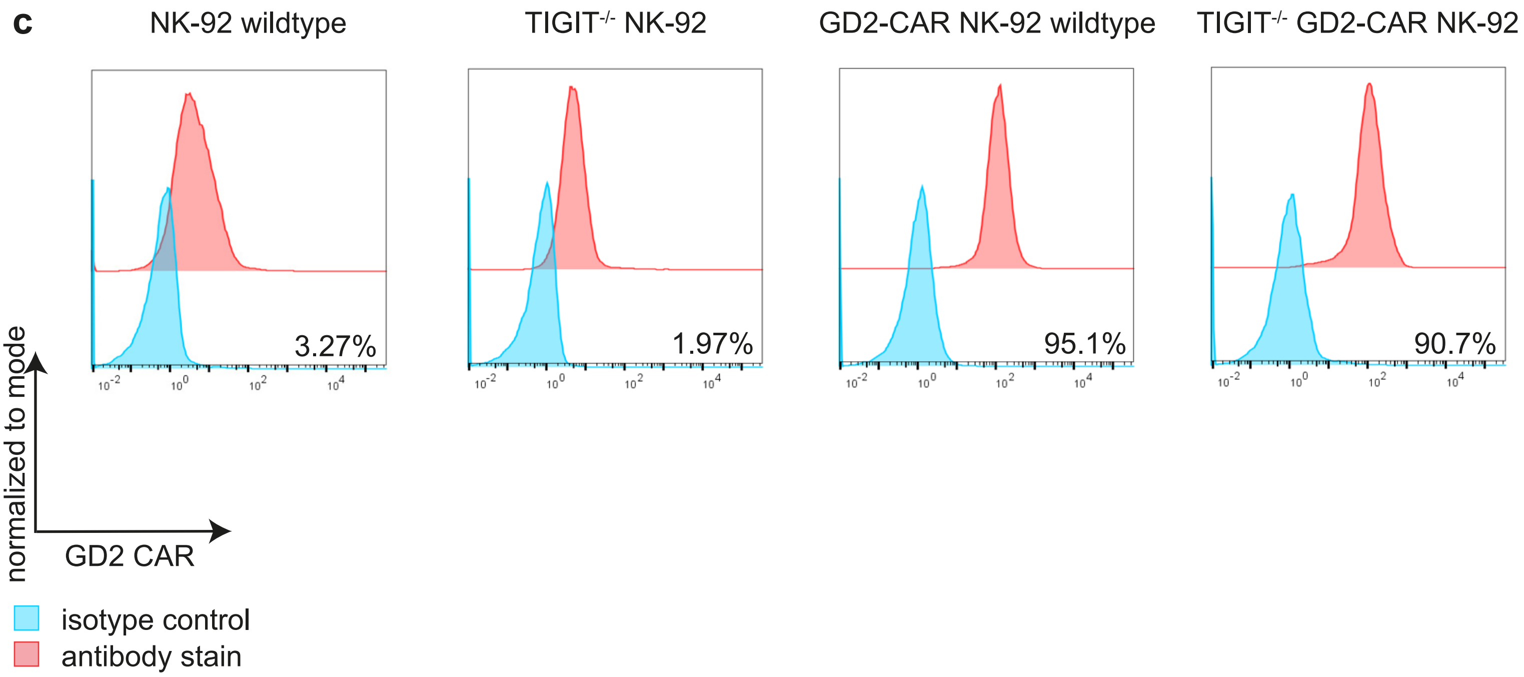

Fig. S5 Generation of TIGIT-/- NK-92, GD2-CAR NK-92 (wildtype) cells and TIGIT-/- GD2-CAR NK-92 cells

a. Flow cytometry screening of TIGIT on NK-92 cells pre and post knock-out. As controls, unstained samples and samples stained using the corresponding isotype were used.
b. Schematic representation of the GD2-CAR vector.
c. Surface staining of transduced NK cells on GD2-CAR expression using biotinylated protein L and Streptavidin-APC/Cyanine 7. As controls, the corresponding isotype was used.

Supplementary Data

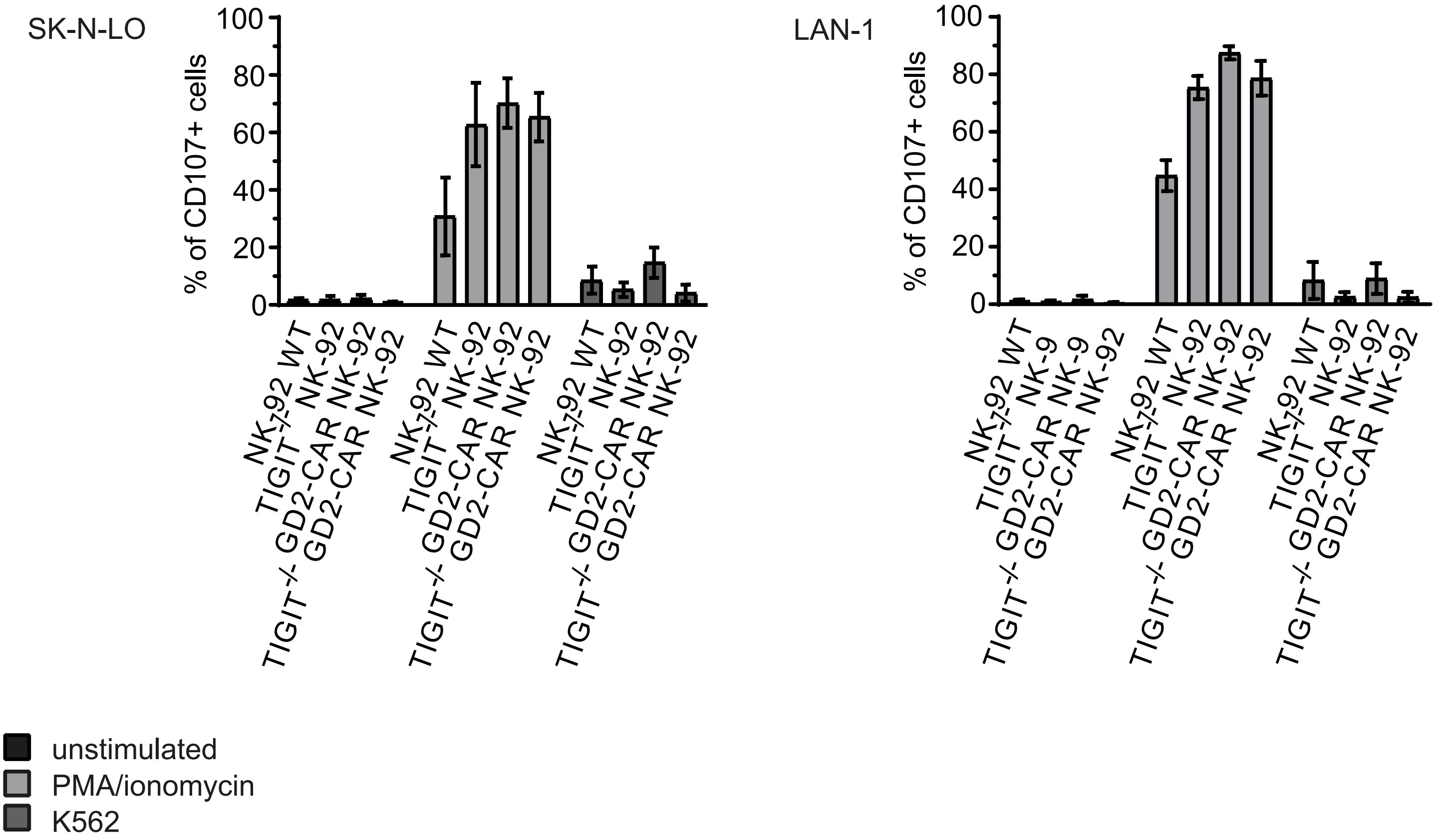

Fig. S6 Controls of degranulation assays using NK-92 effector cells and NB cell lines
Control assays using unstimulated cells, PMA/ionomycin treated cells and NK-92 co-cultured with K562 (n=3). Error bars showing the standard deviation of triplicates of three assays. $p < 0.05$, $p < 0.01$, $p < 0.001$ or $p < 0.0001$ were indicated by *, **, *** or **** respectively.
